# Covering the Distance: A Study of Parent and Teen Attitudes on COVID-19 Mitigation Measures During the COVID-19 Pandemic

**DOI:** 10.7759/cureus.38615

**Published:** 2023-05-05

**Authors:** Jillian Wu, Jane Quinn, Amy B Middleman

**Affiliations:** 1 Pediatrics, Unity Consortium, Philadelphia, USA; 2 Adolescent Medicine, University of Oklahoma Health Sciences Center, Oklahoma City, USA

**Keywords:** covid-19, attitudes, covid-19 vaccines, adolescents, physical distancing, masking, mitigation strategies

## Abstract

Background

The Unity® Consortium surveyed teens and parents and guardians of teens across the country at three distinct time points or waves during the COVID-19 pandemic to assess participant attitudes and beliefs regarding COVID-19 mitigation guidelines, such as mask-wearing and physical distancing.

Methodology

A third-party market research company conducted 15-minute, online surveys from nationally representative panels. Surveys were conducted at three distinct time points or waves (August 2020, February 2021, and June 2021) with 300 teens aged 13-18 years in each wave and 593/531/500 parents and guardians of teens aged 13-18 years in each wave, respectively. Participants responded using a five-point Likert scale (strongly agree to strongly disagree) on their COVID-19 experiences, including the perceived importance of strictly following mask-wearing and/or social distancing guidelines and the perceived effectiveness of mask-wearing and social distancing in preventing the spread of COVID-19. Data were analyzed for differences across waves and demographic variables. Statistical analyses included frequencies, analysis of variance (ANOVA), and t-tests/z-tests.

Results

While significantly more parents and teens in Waves 2 and 3 knew someone who was hospitalized or died due to COVID-19 compared to Wave 1, significantly fewer in Wave 3 reported experiencing a lot or some stress and worry regarding the pandemic. By Wave 3, 58% of teens and 56% of parents had received at least one dose of a COVID-19 vaccine. Despite changes in experiences over time, a significant majority of parents and teens consistently agreed on the importance and effectiveness of social distancing and masking guidelines against the spread of COVID-19. In Wave 3, the demographic variables significantly associated with agreement on importance included race (Black (92%) > White (80%)), community type (urban (91%) > suburban (79%) and rural (73%)), and positive vaccination status of parents and teens (92%/89%) > not vaccinated (73%/73%), respectively). The demographic variables significantly associated with agreement on effectiveness included race (Black (91%) > White (81%)), community type (urban (89%) > suburban (83%) and rural (71%)), and positive vaccination status of parents and teens (94%/90% > not vaccinated (72%/70%), respectively).

Conclusions

This study into the perceived importance and perceived effectiveness of mitigation strategies during the COVID-19 pandemic revealed differences in attitudes among sociodemographic groups. Understanding these differences can help shape how adherence to public health guidelines in a pandemic is promoted.

## Introduction

Since the SARS-CoV-2 virus was detected in the United States, numerous mitigation efforts have been put into place. Initially, as COVID-19 vaccine development began, health authorities of both state and local governments sought to reduce the rate of COVID-19 infections and hospitalizations using non-pharmaceutical interventions (NPIs) [1]. The two most commonly used tactics were the use of masks and physical distancing (commonly referred to as social distancing). Many local and state governments instituted mandates that required face masks or facial coverings over the nose and mouth when inside buildings and/or when outside and in crowded areas where physical distancing was not possible [2-4].

A large body of research has been dedicated to the effectiveness of masking as a mitigation technique during the COVID-19 pandemic, both in the United States and internationally. Studies found that the use of masks reduced infection rates, prevented the spread of viral particles through droplets, and reduced the number of COVID-19 cases [5-7]. One specific meta-analysis evaluating the effectiveness of masking to prevent the spread of SARS-CoV-2 found the infection rate was reduced by nearly 70% among healthcare workers [8]. Furthermore, a general study of the effectiveness of different types of masks found that all mask types limited the dispersal of coughed air, with a focus on mask fit as a factor in minimizing the contaminated field [9].

General attitudes and beliefs surrounding mask-wearing throughout the pandemic have also been studied. Especially early in the pandemic, inconsistency in the adoption of methods based on location, even within the same country or state, exacerbated negative attitudes toward NPIs; low adherence to mask-wearing resulted in increased COVID-19 infections [10]. Mixed messages early in the pandemic from government officials and public health organizations confused the general public on mask-wearing. In the United States, early guidance stating that mask-wearing was not necessary unless around a known infected case was issued to prioritize masks for healthcare providers due to a supply shortage [1]. However, as research established the effectiveness of masking to prevent the spread of COVID-19, recommendations from public health officials seemed inconsistent to the public. Complex and contradictory statements from public figures regarding the severity of the virus and the importance of masking helped fuel anti-masking sentiments in the general public [11]. Anti-masking sentiment and calls to re-open schools, businesses, and public accommodations to pre-pandemic standards may have led some individuals to forego masks completely [12].

Although masking and other non-pharmaceutical mitigation techniques have been proven to be effective methods of limiting COVID-19 spread and infection, sociopolitical factors and individual risk perceptions have influenced individuals’ decisions to wear masks. Studies have found associations between wearing masks and race and ethnicity [12,13]; ideological differences between people from individualistic Western societies such as the United States and collectivist Eastern societies such as China [12]; geographic factors; and socioeconomic status. In addition, adherence has been linked to personal perceptions of risk, including age.

Little is known about the effect of these factors on the attitudes and intentions regarding non-pharmaceutical mitigation strategies among adolescents and their parents. This study aimed to evaluate the attitudes and beliefs regarding mitigation guidelines such as mask-wearing and physical distancing among adolescents aged 13-18 years and among the parents of adolescents over the first 10 months of the pandemic, with Wave 1 starting seven months after the first reported case of COVID-19 in the United States. To help inform the COVID-19 pandemic and in preparation for future pandemics and public health crises, it is important to understand the motivations for these attitudes and beliefs and differences among various communities of young people and their families.

## Materials and methods

**Figure 1:**
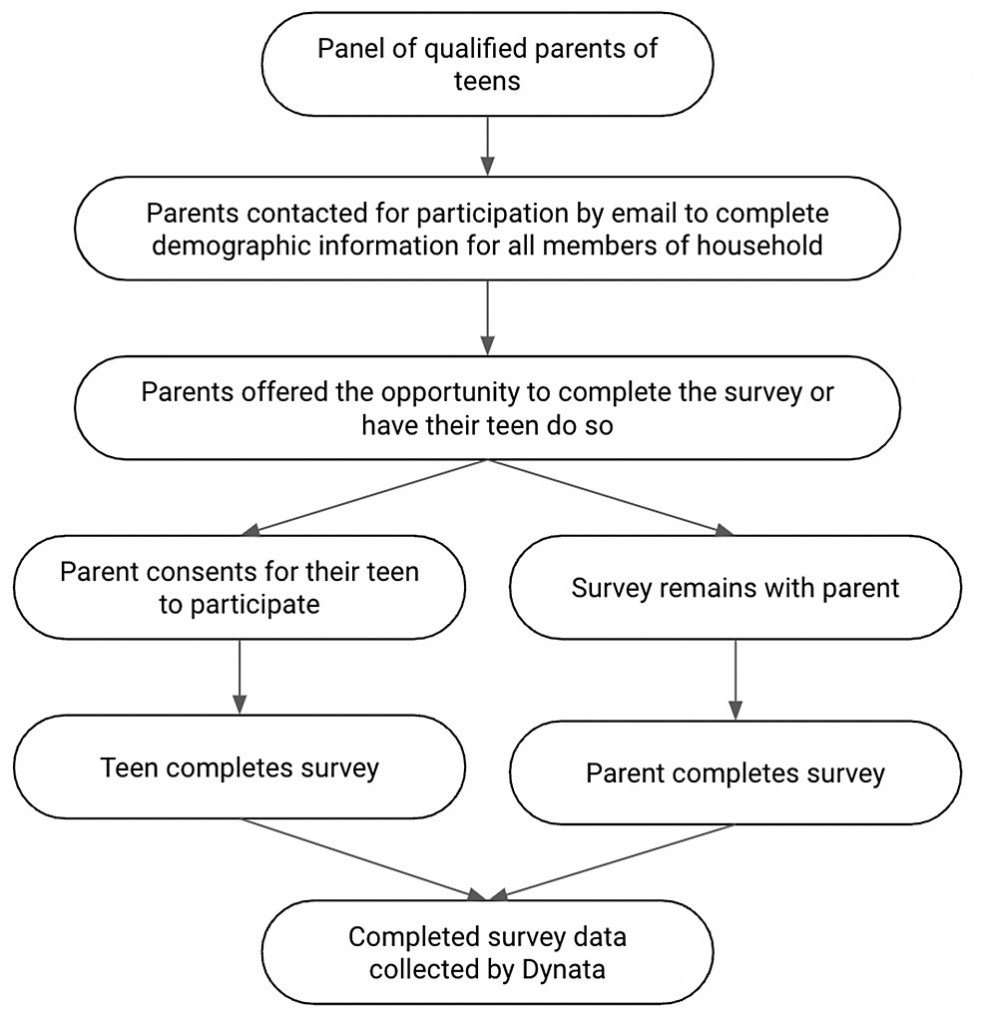
Schematic of the methodology.

A schematic of the study methodology has been outlined in Figure 1.

A survey was developed among members and liaisons of the Unity Consortium, a non-profit organization comprising representatives from academia, medical societies, government and public sector organizations, private sector industry, and numerous immunization, education, and youth advocacy groups working to overcome the barriers to adolescent and young adult (AYA) vaccination. In addition to collecting demographic data, the English-language survey focused on the following three main topics: (1) participants’ experience with COVID-19 and the impact of the pandemic on their lives and activities; (2) attitudes regarding general preventive health behaviors and routine immunizations; and (3) attitudes and intentions regarding COVID-19 prevention, including COVID-19 vaccination. A third-party market research company, Dynata, queried and enrolled qualified participants from existing research panels. Participants, recruited via email, were either the parent or guardian of a 13-18-year-old (who completed the survey with their parent-identified teen in mind) or a parent or guardian willing to consent for their 13-18-year-old to participate in a 15-minute, online, self-administered survey. Parent and teen participants were not related as parent and child dyads. The demographic variables of the teen participants were completed by their parents, and then the teen participants completed the remainder of the survey. Parent participants with more than one child were asked to think of just one of their teen children for their survey responses; the gender and age of the parent-identified teen were noted. Participants were diverse with respect to geography, race, ethnicity, age, education level, and household income. This methodology and sample population have been described in detail previously [14].

Dynata followed all national, regional, and local privacy and data protection laws. Panels complied with all applicable industry standards set by the European Society for Opinion and Marketing Research, Market Research Society (United Kingdom), Australian Market and Social Research Society Limited (Australia), Bundesverband der Marktfoschung (Germany), Insights Association (United States), and other international standards. The study was also approved by the Institutional Review Board at the University of Oklahoma Health Sciences Center.

Surveys were administered at three time points, or waves, during the pandemic, namely, August-September 2020, before COVID-19 vaccine availability; February-March 2021, when COVID-19 vaccine was only recommended for adults aged 18 and older; and June 2021, when COVID-19 vaccines were available and recommended for youth aged 12 and older. All relevant survey items were consistent throughout all three time points, with the exception of additional items related to whether parents (Waves 2 and 3) and teens (Wave 3) had received a COVID-19 vaccine.

Surveys were hosted on a secure website, and all participants were provided a unique link to the questionnaire. Incomplete and partial surveys were excluded. Participant responses were de-identified and other appropriate confidentiality measures were employed throughout the study. Attempts to re-survey participants from prior waves yielded low numbers of repeat participants (39 from Wave 1 in Wave 2; 43 from Wave 2 in Wave 3). This variable was not included in the analyses.

For analysis, demographic variables included gender (male, female, non-binary/other, and prefer not to say) of the parent, parent-identified teen, and teen participant; the age of the parent in years; the age of the teenage child in years; urban/suburban/rural household location; annual household income (<$50,000, $50,000-$99,999, and $100,000+); U.S. Census Bureau region (Northeast, South, West, and Midwest); race (African American, Asian, White, American Indian/Alaskan Native, Native Hawaiian/Pacific Islander, Mixed Race, other, and prefer not to say); and ethnicity (Hispanic and non-Hispanic). Because the sample size of Indian/Alaskan Native and Native Hawaiian/Pacific Islander participants could not support meaningful comparisons in the analysis of the association of response by race, these groups were not represented in the race portion. In addition, items regarding COVID-19 experiences included whether the participant knew a person who either tested positive for, was hospitalized for, or died from COVID-19. How much the participant agreed or disagreed with the following statements was measured using a five-point Likert scale (strongly agree to strongly disagree): I feel a lot or some stress because of COVID-19; I feel safe or very safe going to the store with a mask; and I feel safe or very safe going to the store without a mask. Items regarding mitigation guidelines such as masking and social distancing (i.e., staying 6 feet from others) included how much the participant agreed or disagreed (five-point Likert scale: strongly agree to strongly disagree) with the following statements: It’s very important to follow social distancing and/or mask guidelines as strictly as possible, and Wearing a mask and maintaining social distance (6 feet from others) helps prevent COVID-19 from spreading. Of note, the more commonly used term at the time, social distancing, was included in the survey; the more precise term, physical distancing, is used in this paper. Items also included whether or not the parent, parent-identified teen, or teen participant had been vaccinated (at least one dose) once COVID-19 vaccines became available and recommended for their age group. Proportions reported in the results represent *top box* scores, the sum of the percentages for the two highest points (those who agreed with the statement) on the Likert scale. In this study, it is the sum of the participants who responded with either strongly agree or somewhat agree to survey items.

Data were analyzed by Blueberry Marketing and Sensory Research, a division of Reckner. For Wave 1 only, the participant sample was weighted to account for lower participation from Black respondents. Statistical analyses included frequencies and analysis of variance (ANOVA). If the ANOVA resulted in a significant finding, either a t-test or z-test was used for individual comparisons, as appropriate for the data type. Significant differences were reported at the level of 0.05.

## Results

For Waves 1, 2, and 3, there were 582/300 (weighted), 531/300, and 500/300 parents/teens responding, respectively. The participants are described in Table 1. The demographic proportions of participants described in Table 1 are similar to recently collected census data of the national population in the United States [15]. Of note, the gender of teen participants was relatively similar across waves (Wave 3: 48% female; 51% male; 1% non-binary; 1% prefer not to say).

**Table 1:** Demographic profile of survey participants. *: <0.5%. α: Parent participants were asked to identify one of their teen children about whom to consider when responding to the survey; the gender and age of the parent-identified teen were noted. β: The parent of teen participants completed demographic variables for their children. The teen participants then completed the remainder of the survey.

	Parents/Guardians			Teens		
	Wave 1	Wave 2	Wave 3	Wave 1	Wave 2	Wave 3
Total	582	531	500	300	300	300
Race						
Black or African American	77 | 13%	67 | 13%	70 | 14%	39 | 13%	45 | 15%	45 | 15%
American Indian or Alaskan Native	15 | 3%	2 | <1%	5 | 1%	8 | 3%	2 | 1%	2 | 1%
Asian	15 | 3%	20 | 4%	25 | 5%	8 | 3%	11 | 4%	6 | 2%
White	427 | 73%	396 | 75%	359 | 72%	216 | 72%	225 | 75%	232 | 77%
Native Hawaiian/other Pacific Islander	4 | 1%	4 | 1%	1 | 1%	8 | 3%	0 | 0%	1 | <1%
Mixed race	15 | 3%	19 | 4%	20 | 3%	8 | 3%	8 | 3 %	9 | 3%
Some other race	15 | 3%	19 | 4%	17 | 3%	8 | 3%	6 | 2%	5 | 2%
Ethnicity						
Hispanic origin	33 | 6%	90 | 17%	90 | 18%	31 | 10%	54 | 18%	55 |18%
Not of Hispanic origin	536 | 92%	434 | 82%	407 | 81%	269 | 90%	243 | 90%	245 | 82%
Decline to answer	13 | 2%	7 | 1%	3 | 1%	1 | *	3 | 1%	-
Annual household income						
	138 | 24%	228 | 43%	134 |27%	95 | 32%	113 | 38%	72 | 24%
$50,000–$99,999	173 | 30%	155 | 29%	141 | 28%	76 | 25%	85 | 28%	93 | 31%
>$100,000	247 | 42%	134 | 25%	211 | 42%	107 | 36%	85 | 28%	128 | 43%
Community type						
Urban	199 | 34%	167 | 31%	207 | 41%	109 | 36%	128 | 43%	145 | 48%
Suburban	284 | 49%	242 | 46%	199 | 40%	134 | 45%	120 | 40%	110 | 37%
Rural	99 | 17%	122 | 23%	94 | 19%	57 | 19%	52 | 17%	45 | 15%
U.S. Census Bureau region						
Northeast	109 | 19%	77 | 15%	91 | 18%	79 | 26%	53 | 18%	60 | 20%
South	215 | 37%	202 | 38%	193 | 39%	98 | 33%	121 | 40%	94 | 31%
Midwest	125 | 22%	124 | 23%	103 | 21%	53 | 18%	60 | 20%	65 | 22%
West	133 | 23%	128 | 24%	113 | 23%	71 | 24%	66 | 22%	81 | 27%
Positive vaccination status						
Yes	-	-	288 | 58%	-	-	176 | 59%
Age						
Mean (years)	45.8	44.1	45.5	-	-	-
Ages 25–34	34 | 6%	34 | 6%	33 | 7%	-	-	-
Ages 35–44	251 | 43%	282 | 53%	223 | 45%	-	-	-
Ages 45–54	203 | 35%	160 | 30%	159 | 32%	-	-	-
Ages 55–64	88 | 15%	42 | 8%	72 | 14%	-	-	-
Ages 65 and older	6 | 1%	13 | 2%	13 | 2%	-	-	-
Gender	Parent/Guardian			Parent-identified teen^α^		
Female	314 | 54%	354 | 67%	285 | 57%	183 | 61%	175 | 58%	162 | 54%
Male	264 | 45%	176 | 33%	215 | 43%	114 | 38%	125 | 42%	138 | 46%
Non-binary/Other	-	-	-	1 | *	-	-
Prefer not to say	4 | 1%	1 | *	-	2 | 1%	-	-

COVID-19 experiences

The percentage of parent and teen participants who reported knowing someone who tested positive for COVID-19, was hospitalized due to COVID-19, or died from COVID-19 significantly increased between Waves 1 and 2 and was consistent between Waves 2 and 3. The percentage of parents and teens who felt a lot or some stress because of COVID-19 remained consistent between Waves 1 (72%/67%) and 2 (76%/68%) but significantly decreased for both parents and teens in Wave 3 (65%/57%). There was a significant increase in the proportion of parents and teens who felt very safe either going to the store with a mask or going to the store without a mask in Wave 3 compared to Waves 1 and 2 (Table 2).

**Table 2:** COVID-19 experiences of survey participants. Note: An upper-case letter indicates a significant difference (p < 0.05) in proportion from the column indicated by the letter.

	Parents/Guardians			Teens		
	Wave 1 (A)	Wave 2 (B)	Wave 3 (C)	Wave 1 (D)	Wave 2 (E)	Wave 3 (F)
I know someone...						
who tested positive for COVID-19	38%^D^	65%^AC^	59%^AF^	29%	52%^D^	46%^D^
who was hospitalized due to COVID-19	16%	28%^A^	31%^A^	15%	25%^D^	25%^D^
who died from COVID-19	13%	23%^A^	26%^AF^	10%	22%^D^	18%^D^
I feel a lot or some stress because of COVID-19	72%^C^	76%^CE^	65%^F^	67%^F^	68%^F^	57%
I feel safe or very safe going to the store with a mask	62%	62%	77%^AB^	66%	63%	80%^DE^
I feel safe or very safe going to the store without a mask	26%^B^	18%	34%^AB^	22%	22%	33%^DE^

Importance of adhering to mitigation strategies

**Figure 2:**
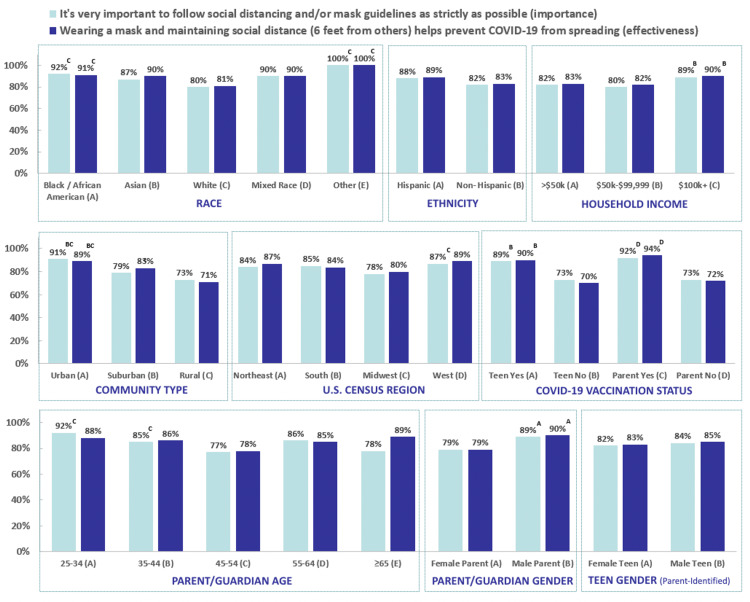
Reported agreement of all survey participants with social distancing/mask statements by demographic variables and COVID-19 vaccination status (Wave 3). Notes: Top 2 box responses (strongly agree/somewhat agree) are reported. An upper-case letter indicates a significant difference (p < 0.05) in proportion from the column indicated by the letter.

Survey responses of strongly or somewhat agree that following mitigation strategies such as social distancing and/or masking as strictly as possible is important to prevent COVID-19 spread are presented in Figure 2. Across the three waves, over 80% of parents and teens strongly or somewhat agreed with the statement, with no significant differences among waves. When comparing the responses in Wave 3 by race, ethnicity, annual household income, community type, geographic region, COVID-19 vaccination status, parent age, parent gender, and gender of the parent-identified teen, the variables associated with agreement were race, community type, COVID-19 vaccination status, and parent gender. In Wave 3, Black respondents (92%) were significantly more likely than White respondents (80%), urban households (91%) were significantly more likely than suburban (79%) and rural (73%) households, vaccinated parents (92%) and vaccinated teens (89%) were significantly more likely than unvaccinated parents (72%) and unvaccinated teens (73%), and male (89%) more likely than female (79%) parents to strongly or somewhat agree that following mitigation strategies as strictly as possible was important. Interestingly, parent gender was not associated with the top box response rate in Waves 1 and 2. While there were some statistically significant differences in response based on parental age category in Wave 3, there was no identifiable trend.

Effectiveness of mitigation strategies

Survey responses of strongly or somewhat agree that mitigation strategies such as social distancing and/or masking are effective for preventing COVID-19 spread are presented in Figure 2. Across the three waves, over 80% of parents and teens strongly or somewhat agreed with the statement, with no significant differences among waves. When analyzing associations of survey responses in Wave 3 by race, ethnicity, annual household income, community type, geographic region, COVID-19 vaccination status, parent age, parent gender, and gender of the parent-identified teen, the variables of race, household income, community type, geographic region, vaccination status, and parent gender were found to be significantly associated with agreement. Black participants (91%) were more likely than White participants (81%) and male parents (90%) were more likely than female (79%) parents to strongly or somewhat agree that masking and physical distancing were effective in preventing COVID-19 spread. Households making over $100,000 annually (90%) were more likely than households making between $50,000 and $99,999 annually (82%) and households making less than $50,000 annually (84%) to agree that masking and physical distancing were effective strategies; urban households (89%) were more likely than suburban households (83%) which were more likely than rural (71%) households and households in the West (89%) were more likely than Midwestern households (80%) to agree. Vaccinated parents (94%) and teens (90%) were more likely than unvaccinated parents (72%) and teens (70%) to strongly or somewhat agree that masking and physical distancing were effective in preventing the spread of disease. Again, parent gender was not associated with the top box response rate in Waves 1 and 2.

## Discussion

This study is the only one of which we are aware that has explored the attitudes of teens and parents of teens regarding masking and physical distancing at three distinct time points (or waves) during the first 18 months of the COVID-19 pandemic. Over the course of the pandemic, scientific studies have validated the effectiveness of masking and physical distancing to slow the spread of disease via modeling research as well as analyses of data collected from multiple metropolitan areas that experienced mask mandates versus no mask mandates [2,9,16-18]. Ultimately, the data from this survey support that the majority of teens and the parents of teens (>80% of teens and parents) either strongly agreed or agreed with the importance and effectiveness of these NPI strategies to prevent the spread of COVID-19. These attitudes did not waver despite changing rates of knowing someone who was hospitalized or died due to COVID-19, rates of reported stress or safety during the pandemic, or even availability and rates of vaccination over the course of the three survey waves.

Although existing studies have not necessarily tracked the attitudes of teens and teens’ parents over time during the pandemic, other investigators have measured attitudes and adherence to these NPIs among adults during the pandemic. Findings from an international study on perceived usefulness and adherence to mitigation measures revealed that among adults responding to an online survey from eight different countries (United Kingdom, Spain, France, Germany, United States, Poland, Russia, and Sweden), poor adherence to mitigation strategies was associated with perceived ambivalence of government policy [19]. Factors positively associated with adherence included female gender, higher age, belonging to a risk group, being affected economically/mentally/physically by the pandemic, feeling well informed, and trusting that the government was guided by the people’s interests [19]. In a review of 29 studies, Moran et al. also found that the female gender, older individuals, those who trust the government, and those who perceive COVID-19 as a threat to health were more likely to adhere to COVID-19 public health guidelines [20]. A perceived threat to health by COVID-19 has been consistently associated with positive attitudes toward NPIs [1,19,21,22]. In one of the few studies that followed adult behaviors over time during the pandemic, national surveys from 16 waves of the Coronavirus Tracking Survey in the United States found that between April and November 2020 in the United States, the NPI adherence index (number of NPI behaviors reported in the week before the survey adjusted for sociodemographic factors) dropped significantly in all U.S. Census regions; however, specifically wearing a mask or a face covering increased during this time period from 39% to 89% among respondents [23]. This finding of decreased adherence to physical distancing and isolation coupled with an increase in mask-wearing in the first year of the pandemic, especially among younger adults, seen in some studies implies a certain degree of burnout related to mitigation strategies and perhaps a willingness to trade social isolation for increased mask adherence [24]. In our study, while adherence per se was not measured, attitudes regarding the importance and effectiveness of NPIs remained relatively consistent among both young people and the parents of young people across the first 18 months of the pandemic. Because we did not examine individual NPIs, it is possible the consistency seen in our study represents no net change between the alternating importance of masking versus being able to increase social activity that is so important among younger people.

This study identified a number of sociodemographic variables associated with a greater likelihood of perceived importance and effectiveness of masking and physical distancing among teens and parents of teens during the pandemic. By Wave 3 of the survey, variables positively associated with the agreement that following mitigation strategies as strictly as possible was important were race (Black/African American compared to White), male parent gender, community type (urban compared to suburban and rural), and positive vaccination status. No significant difference was associated with ethnicity, gender of the parent-identified teen, household income, or household region. Variables positively associated with the agreement that mitigation strategies were effective were race (Black/African American compared to White), household income ($100k+ compared to <$50k and $50k-$99,999), community type (urban compared to suburban and rural; suburban compared to rural), household region, positive vaccination status, and male parent gender. Ethnicity was not associated with any significant differences in agreement.

Overall, the proportion of Black respondents who agreed that following mitigation guidelines was important and that mitigation guidelines were effective was significantly greater than the proportion of White respondents who agreed. This may be because the COVID-19 pandemic has disproportionately affected communities of color, especially Black respondents who often have less access to healthcare, are more likely to be frontline or essential workers, and deal with historic and systemic adverse social determinants of health [25-28]. Masking and physical distancing are much less invasive strategies compared to vaccination, about which there is historic medical mistrust and potential inequities related to healthcare access and service [29,30].

Additionally, participants with greater wealth were significantly more likely to agree with the effectiveness of masking and physical distancing. During the pandemic, the ability to purchase a mask in person or online or have multiple masks for everyone in the household may have been an issue of financial capacity. Those with lower incomes may have a more difficult time masking or physical distancing during the pandemic, especially if they work in an essential, public-facing job [12]. Other studies have shown that those of lower socioeconomic or educational status were more likely to have misconceptions regarding the virus and transmission [31].

Furthermore, urban residents were significantly more likely to agree with the importance and effectiveness of masking and physical distancing than suburban and rural residents. This may be due to the differing population density and the increased need to mask and maintain physical distance in an urban setting compared to suburban or rural settings where more options to quarantine and/or shelter in place may exist. Historically, pandemics and crises impact marginalized populations disproportionately, further exacerbated by an urban environment where very high density, unequal access to basic services, and high cost of living make it difficult for many residents to practice physical distancing, quarantine, or follow stay-at-home orders [32]. Interestingly, in Wave 3, suburban residents were also significantly more likely to agree on the effectiveness of masking and physical distancing compared to rural residents. Furthermore, participants from the West were significantly more likely than participants from the Midwest to agree with the effectiveness of masking and physical distancing. This may be due to variability in state policies and warrants further study.

To our knowledge, this is the first study to examine the association between attitudes regarding NPIs and vaccination status. Positive vaccination status was associated with agreement on the importance and effectiveness of mitigation strategies for both teens and parents. This association was only studied in Wave 3 after COVID-19 vaccines were made available to teens. This association makes sense, as those who trust in or are willing to get vaccinated are likely also those who trust in or are willing to follow mitigation guidelines. Anti-vaccination messaging during the COVID-19 pandemic has been mixed with anti-masking messages, which may explain the significantly lower rates of agreement with NPI importance and effectiveness among parents and teens who are anti-vaccine or vaccine-hesitant.

This study did not note major differences based on parent age; it is important to note that this variable was skewed to people in the younger age categories. It is not clear why male parents were more likely to agree with the importance and effectiveness of NPIs by Wave 3 when most other studies describe females as being more likely to engage in such strategies. This finding warrants further investigation.

There are several limitations to this study. The study was conducted using research panels consisting of people interested in research, potentially creating a selection bias. This is demonstrated by the vaccination rate of 58% reported for teens by June 2021 in this study; national Centers for Disease Control and Prevention data indicate that as of July 31, 2021, 42.4% of adolescents aged 12-17 years have had more than one COVID-19 vaccine [33]. In addition, the survey was only available to those who can read English. Furthermore, the number of Native American/American Indian or Native Hawaiian/Pacific Islander participants did not provide the power for meaningful comparison. The parent/guardian participants were not related to the teen participants, eliminating the ability to note possible concordance within parent-child dyads. These data were collected before the emergence of the SARS-CoV-2 Delta and Omicron variants; with increased rates of disease associated with these variants, the perceived importance and effectiveness of mitigation strategies may have shifted. Finally, ideally, the survey would have assessed adherence to NPIs as well as attitudes, understanding that attitudes are not always predictive of behavior; behavior variables were not included given the inability to validate such data.

In summary, despite variable COVID-19 and vaccination experiences, attitudes and beliefs related to mitigation strategies for adolescents were positive across survey waves. There were differences in the degree of agreement that physical distancing and masking are important and effective across several demographic variables. Black respondents, urban residents, and vaccinated respondents were significantly more likely to agree with the importance of masking and physical distancing; Black respondents, urban residents, higher-income households, and vaccinated respondents were significantly more likely to agree that masking and physical distancing were effective. This study is unique in its exploration of the perceived importance and effectiveness of mitigation strategies across a 10-month continuum of the COVID-19 pandemic among parents of teens and teens themselves. Understanding differences in attitudes among sociodemographic groups can help shape and target future messaging to promote and support adherence to public health guidelines in a pandemic environment, now and in the future. Research using the same survey found that Black teens and White teens were vaccinated at similar rates [14], yet Black respondents were significantly more likely than White respondents to agree with the perceived importance and perceived effectiveness of mitigation strategies. Attitudes and beliefs related to mitigation strategies did not follow the same patterns of association as vaccination behaviors. It will be critical to understand to whom to target specific messaging for both mitigation and vaccination strategies as both public health approaches will be required to curb the spread of disease in any future pandemics.

## Conclusions

This study into the perceived importance and perceived effectiveness of mitigation strategies during the COVID-19 pandemic revealed differences in attitudes among sociodemographic groups. Adolescent attitudes and beliefs toward mitigation strategies such as masking and physical distancing were consistently positive across survey waves. Understanding these differences can help shape how adherence to public health guidelines during a pandemic is promoted. Furthermore, understanding public health messaging for mitigation and vaccination strategies is a critical next step in combating future pandemics.
